# Distinct adhesion energy and extracellular matrix profiles of healthy and osteoarthritic human chondrocytes

**DOI:** 10.1007/s11626-026-01193-9

**Published:** 2026-06-11

**Authors:** Robert Stannert, Lindsey Attebery, Abraham Rodriguez

**Affiliations:** 1 University of Texas San Antonio, San Antonio, TX USA; 2 UT Health San Antonio, San Antonio, TX USA; 3 Department of Biomedical Engineering and Chemical Engineering, Klesse College of Engineering and Integrated Design, The University of Texas at San Antonio (UTSA) & UT Health San Antonio, San Antonio, TX USA

**Keywords:** Osteoarthritis, Chondrocytes, Adhesion energy, Collagen, Glycosaminoglycans

## Abstract

Osteoarthritis (OA) is a common degenerative joint disease characterized by the progressive degeneration of articular cartilage (AC), resulting in pain, stiffness, and reduced joint function. Despite its widespread prevalence, effective treatments for OA remain elusive, partly due to challenges in accurately modeling the disease. In the present study, adhesion energy, collagen (COL), and total glycosaminoglycans (GAG), the main extracellular matrix (ECM) components of AC, and their potential correlations were evaluated in human chondrocytes derived from both healthy and OA male donors. A model surface of silicon nitride was used to investigate adhesion energy using atomic force microscopy (AFM), while colorimetric assays were applied to quantify COL and GAG at five different time points during monoculture (days 0, 1, 7, 14, and 21). Our results indicate significant heterogeneity in adhesion energies at both donor and phenotype levels. Chondrocytes isolated from healthy donors consistently display higher adhesion energies, along with greater heterogeneity, compared to those derived from OA donors. These differences in adhesion energy and heterogeneity can be attributed to greater diversity and density of cellular surface molecules in cells from healthy donors. In contrast, total GAG and COL levels were significantly elevated in OA chondrocytes compared to those in healthy chondrocytes. Moreover, the study found a positive correlation between adhesion energy and GAG production in OA-derived chondrocytes, but no significant correlations with DNA or COL levels. No correlation was observed between adhesion energy and GAG, DNA, or COL levels in healthy cells. In conclusion, these findings highlight the importance of considering individual donor characteristics when developing OA disease models, therapeutics, or engineered tissues.

## Introduction

Osteoarthritis (OA) is the most prevalent degenerative disease, affecting over 33 million individuals in North America [1]. According to the Centers for Disease Control and Prevention (CDC), the number of individuals aged 65 and older affected by OA is expected to nearly double [2]. This increase is estimated to rise from 21.4 million cases in 2005 to 41.1 million by 2030 [2]. OA can affect all synovial joints; it occurs when the protective articular cartilage (AC), which covers the ends of bones in movable joints, undergoes progressive wear and degradation [3]. Management of OA often includes lifestyle modifications such as physical therapy, exercise, or weight loss, while severe cases may require total joint replacement despite its invasive nature and high cost [4–6]. The absence of effective treatments is partly attributed to the lack of appropriate disease models that accurately capture OA phenotypes and enable the evaluation of potential therapeutic interventions.

Current models of OA struggle to balance human anatomical relevance with experimental feasibility [7]. The scarcity and rapid dedifferentiation of chondrocytes, particularly from OA donors, complicate optimization of outcomes [8]. While cells derived from healthy tissue offer better proliferation and phenotypic stability, OA-derived cells, despite their limited availability and slower growth, provide more clinically relevant models for therapeutic trials [9, 10].

The two main components of AC are chondrocytes, the predominant cell type within AC tissues, and the extracellular matrix (ECM), a gel-like substance primarily composed of water, collagen (COL), and proteoglycans [11]. They participate in the synthesis and maintenance of the ECM and facilitate the growth and repair of healthy AC through diffusion across a concentration gradient [12, 13]. COL is a structural protein that provides tensile strength to the cartilage ECM. In contrast, proteoglycans are complex molecules spread throughout the ECM, composed of proteins covalently attached to glycosaminoglycans (GAG). Cellular adhesion energy is also an essential factor for tissue formation. Cell adhesion and surface interactions between chondrocytes and surrounding surfaces play a critical role in regulating cellular behavior, including cell proliferation, migration, cytoskeletal organization, and ECM synthesis. In AC, these adhesive interactions contribute to the structural stability of the tissue and influence how chondrocytes sense and respond to their mechanical microenvironment. Changes in cartilage composition during OA, such as degradation of the COL network and changes in GAG content, can alter the physicochemical properties of the ECM and potentially affect cell adhesive behavior. At the molecular level, adhesive interactions between key components of the ECM, such as aggrecan and COL, have been shown to play a role in the structural organization of cartilage and may be altered during degenerative processes associated with OA. Advances in nanoscale biomechanical techniques, particularly atomic force microscopy (AFM), have enabled direct measurements of mechanical properties and cell adhesion with high spatial resolution, providing valuable insights into cartilage biology and OA progression. AFM-based force spectroscopy has been successfully used to quantify the adhesion forces of living chondrocytes and to investigate cell–substrate interactions during cell attachment and spreading. Despite these advances, limited studies have directly compared the adhesion energies of healthy and arthritic human chondrocytes and examined how these mechanical interactions are related to ECM production. Therefore, a better understanding of the relationship between adhesion energy and ECM synthesis in healthy versus arthritic chondrocytes could provide new insights into cellular functional differences associated with OA and improve the development of physiologically relevant laboratory models.

In the present study, isolated chondrocytes from three healthy donors and three age-matched donors with OA were cultured in a monolayer for 21 d. Samples were evaluated colorimetrically for GAG and COL content. In addition, the adhesion energy of the cells to a silicon nitride model surface was measured using AFM. Finally, potential correlations between the calorimetric results and the measured adhesion energies were investigated.

## Materials and methods

### Cell culture

Human chondrocytes isolated from three healthy male individuals (CELLvo™ Healthy Human Chondrocytes, passage 0, age 62.3 ± 2.1 yr) and three male individuals with OA (CELLvo™ Osteoarthritic Human Chondrocytes, passage 0, age 64.6 ± 1.7 yr) were generously provided by StemBioSys, Inc. (San Antonio, TX). The donors were between 50 and 70 yr old. Cells were isolated from human AC (knee joint, as specified by the supplier). Healthy donors had no reported history of OA, while donors with OA were clinically diagnosed with OA. Detailed information on disease severity (e.g., OA severity) was not provided by the supplier. Cells were frozen at passage 0 and expanded before use. There was no significant difference in donor age between groups (*P* = 0.1687). Following cell expansion, the cells (at passage 2) were cultured at a density of 9 × 10^3^ per well in 48-well plates for colorimetric assays, and at a density of 3.6 × 10^4^ cells in 35-mm Petri dishes for AFM experiments. Cells were cultured under DMEM/F-12 (Invitrogen, Carlsbad, CA) supplemented with 10% v/v FBS, 1% v/v Pen-Strep, and 1% v/v Fungizone (All from Sigma Aldrich, St. Louis, MO) and incubated at 37°C in 5% CO_2_, 95% relative humidity for 0, 1, 7, 14, and 21 days.

### Colorimetric quantification of COL and GAG

Engineered AC tissues were evaluated for ECM production by measuring COL and GAG contents, normalized by DNA on days 0, 1, 7, 14, and 21. For DNA extraction, cells were digested overnight in a papain extraction reagent aqueous buffer suspension (0.1 mg/mL papain (≥ 16 units/mg protein) in 0.2 M sodium phosphate/ethylenediaminetetraacetic acid (EDTA)) (all from Sigma Aldrich) at 65°C. DNA was quantified using the Quant-iT Pico Green dsDNA Assay Kit (Invitrogen), following the manufacturer’s instructions [14]. Fluorescence was read at excitation and emission wavelengths of 480 nm and 520 nm, respectively, using a Cytation-5 Microplate reader (Biotech instruments, Winooski, Vermont). COL and GAG were quantified for cells using the Sircol (soluble) and Blyscan assays kits (Biocolor Ltd., Belfast, UK), respectively. Following the manufacturer’s protocols. COL and GAG optical densities were measured at 555 nm and 656 nm, respectively, using the Cytation-5 microplate reader. Both COL and GAG contents were normalized to DNA (ng) and are reported in micrograms. All measurements were performed in triplicate.

### AFM measurements

AFM imaging and adhesion measurements were conducted on cells representing different time points during the cell culture process, on days 0, 1, 7, 14, and 21, utilizing the Nano-Wizard IV AFM (JPK Instruments, Bruker, Billerica, MA) coupled with the Cell-Hesion module. The later module allows for large vertical piezo-element displacements (≤ 100 µm) to be measured. The AFM head is mounted on a Zeiss Axio Observer 3 microscope (Carl Zeiss, Göttingen, Germany). All measurements were taken in PBS at 37°C using a temperature-controlled Petri-dish heater. Non-conductive silicon nitride tips with 18-kHz resonance frequency and a nominal spring constant of 0.06 N/m (DNP-10, probe D, Bruker) were used. Cantilever spring constants were calibrated individually using the thermal oscillation method [15]. The QI™ Imaging mode was used to acquire the AFM height images of individual cells at a resolution of 128 × 128 pixels, with a speed of 300 µm/s, and a set point of 2 nN to avoid damaging the surfaces of cells during scanning [16]. Following the acquisition of a topographic image of a specific cellular entity, the force-mapping mode was implemented to impose an 8 × 8 grid onto the cellular region. Force–displacement curves were recorded across the entire cell body, with a maximum applied load of 3 nN, a *Z*-length of 15 µm, and a speed of 5 µm/s. For each donor, three separate cultures were prepared, and three cells representing each culture were randomly probed, yielding 64 curves per individual cell for analysis.

**Figure 1.**
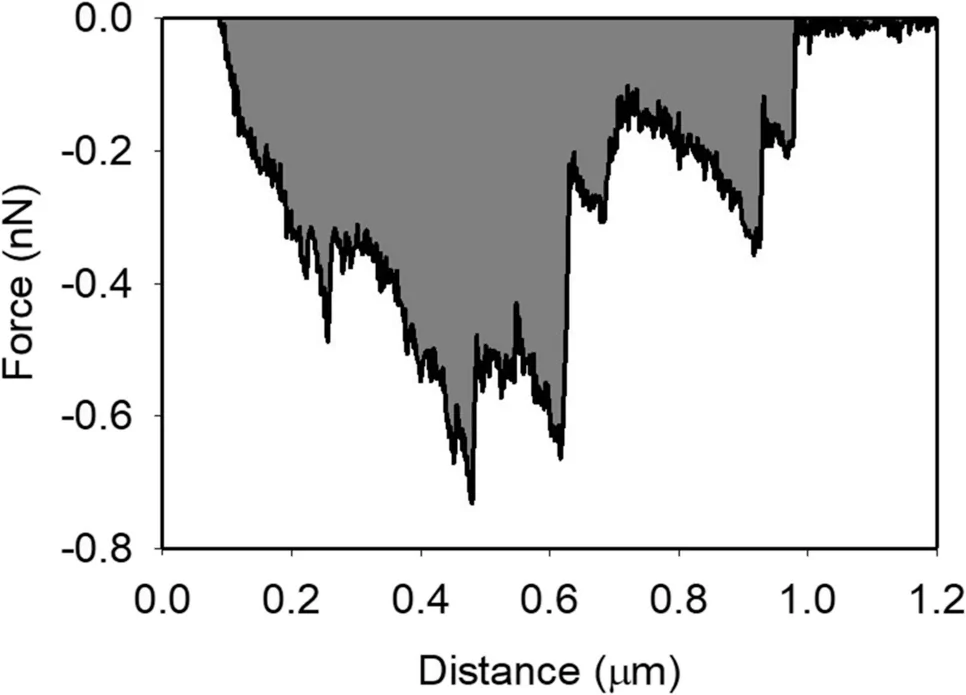
Example of a retraction curve. The *shaded area* under the curve represents the adhesion energy.

Using the AFM machine software, retraction curves were individually analyzed to quantify the adhesion energy measured between the surface of a human chondrocyte cell and a silicon nitride tip in phosphate-buffered saline (PBS, Invitrogen). The adhesion energy was calculated as the area under the force-distance retraction curve, with the baseline set at zero force (Fig. 1). High-quality curves were used in this paper, selected based on factors such as smoothness, absence of noise, and consistency of the baseline.

### Statistics

All experiments were performed independently at five time points using three separate donors for both healthy and diseased groups (*N* = 3), with three technical replicates per condition (*n *= 3). Statistical analysis was conducted using linear mixed models (LMM) in jamovi (v2.3.2), utilizing the R environment to account for the hierarchical data structure. In the LMM, phenotype (healthy vs. OA) and time points were treated as fixed effects, while donor identity was included as a random effect to account for biological variability and the non-independence of repeated measurements. This approach allows for the simultaneous modeling of experimental factors and random variation from biological and technical sources. Model assumptions were verified via residual diagnostics, including *Q*-*Q* plots and homoscedasticity tests. Statistical significance was set at *P* < 0.05. Data were visualized using box plots (displaying median, interquartile range, and outliers) or mean plots where error bars indicate the standard error (SE) of the estimated marginal means derived from the model. Correlations between adhesion energy and biochemical markers were evaluated via linear regression (*R*^2^).

## Results and discussion

### Adhesion energies - Donor to donor variability

**Figure 2.**
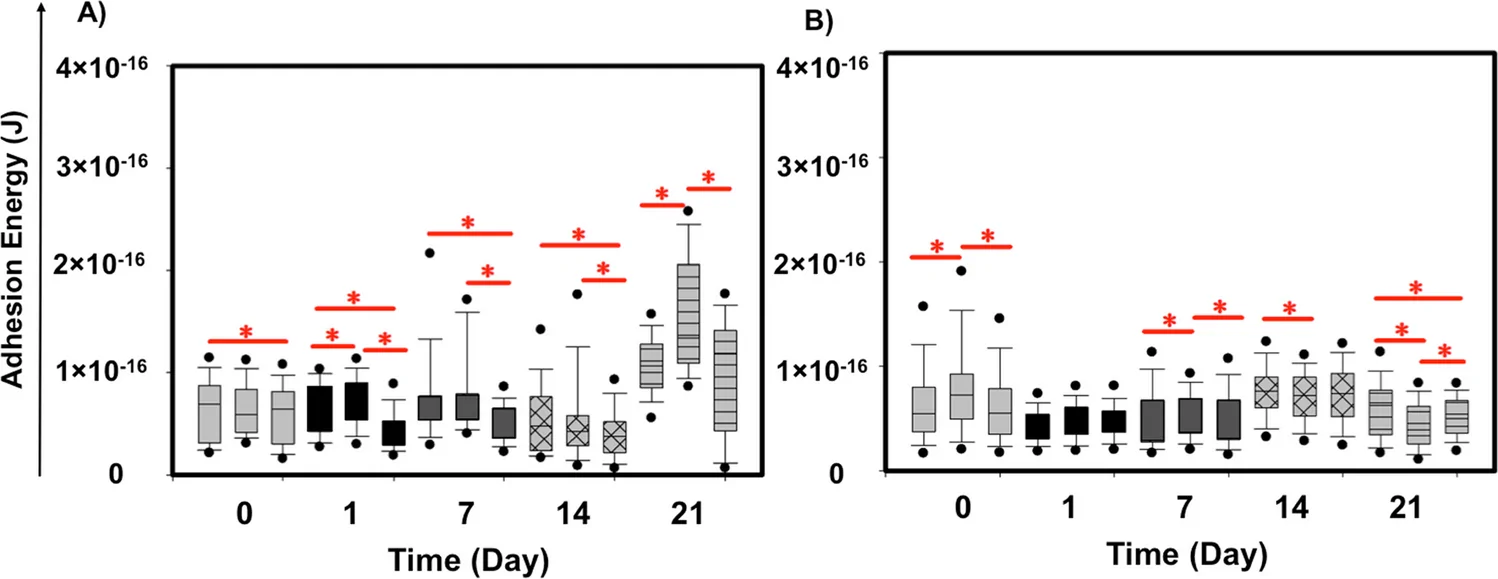
Adhesion energies of human chondrocytes on a silicon nitride surface. Adhesion energy measurements for (*A*) healthy and (*B*) osteoarthritis (*OA*) chondrocytes were conducted in buffer at 37°C across five time points (days 0, 1, 7, 14, and 21). Data are presented as box plots (median, interquartile range, and individual data points). The estimated marginal means ± standard error (SE) were derived from the linear mixed model (LMM). The model accounts for the hierarchical structure of the dataset, including three independent donors per group (*N* = 3) and three separate cultures per donor (*n* = 3). *Error bars* reflect the uncertainty in the estimated fixed effects after accounting for donor-to-donor variability as a random effect. *One asterisk* indicates significance (*P* < 0.05).

To investigate donor-to-donor variability in adhesion energies, a total of 27 cells from each donor were mapped, resulting in between 502 and 576 data points for each sample using AFM (Table 1). As shown, regardless of whether the cells were from healthy (Fig. 2*A*) or OA (Fig. 2*B*) donors, the data revealed significant variability across cultures derived from the same donor at the same time point. Specifically, all data from the three healthy donors showed considerable variation in adhesion energies at all time points. Similarly, significant variation in adhesion energies was detected across all time points in the OA donor cells, except on day 1, where no significant differences were observed.

**Table 1.** Mean adhesion energies (E; × 10^–17^ J) for individual donors from healthy (*H*) and osteoarthritis (*OA*) groups

		Day 0 (10^–17^ J)	Day 1 (10^–17^ J)	Day 7 (10^–17^ J)	Day 14 (10^–17^ J)	Day 21 (10^–17^ J)
H	*Donor 1*	6.5 ± 3.1	6.5 ± 2.5	7.7 ± 5.1	5.8 ± 4.9	10.8 ± 3.5
	*Donor 2*	6.4 ± 2.7	7.0 ± 2.6	8.1 ± 4.3	6.0 ± 7.1	15.6 ± 6.1
	*Donor 3*	6.0 ± 3.0	4.4 ± 2.8	5.1 ± 2.0	4.4 ± 4.4	10.1 ± 5.5
OA	*Donor 1*	6.5 ± 4.1	4.3 ± 1.6	5.1 ± 3.3	7.6 ± 2.6	6.0 ± 2.8
	*Donor 2*	8.1 ± 5.1	4.8 ± 2.0	5.3 ± 2.3	7.1 ± 2.7	4.3 ± 2.2
	*Donor 3*	6.3 ± 4.1	4.7 ± 1.7	5.1 ± 3.1	7.3 ± 2.9	5.1 ± 2.1

Values are reported as mean ± standard deviation

Furthermore, chondrocytes from healthy and OA donors demonstrated differences in their adhesion energies at a constant time point. On day 0, the values for chondrocytes derived from healthy donors ranged from 6.0 ± 3.0 × 10^–17^ to 6.5 ± 3.1 × 10^–17^ J. The differences in adhesion energy among isolated cells from healthy donors persisted on days 1, 7, and 14, with the highest values being 7.0 ± 2.6, 8.1 ± 4.3, and 6.0 ± 7.1, and the lowest values being 4.4 ± 2.8, 5.1 ± 4.3, and 4.4 ± 4.4, respectively. On day 21, noticeable variability was observed, with the highest value being 15.6 ± 6.1 and the lowest 10.1 ± 5.5.

In OA-derived chondrocytes, variability was similarly detected, with the highest values on days 0, 1, 7, 14, and 21 being 8.1 ± 5.1, 4.8 ± 2.0, 5.3 ± 2.3, 7.6 ± 2.6, and 6.0 ± 2.8, respectively, while the lowest values were 6.3 ± 4.1, 4.3 ± 1.6, 5.1 ± 3.1, 7.1 ± 2.7, and 4.3 ± 2.2 (Table 1).

These findings indicate significant donor-level variability, which was explicitly accounted for in statistical analyses using a linear mixed model including donor identity as a random effect. Donor variability likely reflects biological heterogeneity in genetic background and cellular phenotype. Each donor has a unique genetic makeup that influences cell adhesion properties. Variations in integrin expression, cytoskeletal components, and ECM production influence how cells adhere to surfaces. For example, one study discovered significant differences in chondrogenic potential among human mesenchymal stem cells (hMSCs) from different donors after culture in 3D hyaluronic acid (HA) hydrogels, exhibiting that donor-specific factors affect cell behavior [17]. Several other factors, such as ethnicity, gender, and age of the donor, may also influence outcomes. A study of 14 donors indicated that age may significantly impact the chondrogenic redifferentiation capacity of cells isolated from human AC. These intrinsic donor characteristics affect cell adhesion and differentiation outcomes [18]. Another study (in vitro) investigated the effect of extracellular calcium ion concentration on isolated human chondrocytes and found that these ions regulate cell migration, chondrocyte stiffness, and ECM cell adhesion. Their results showed that increasing calcium ion concentration led to higher elastic modulus and increased cell–ECM adhesion forces, possibly due to changes in the expression of myosin II and integrin β1 [19].

### Phenotype variability

**Figure 3.**
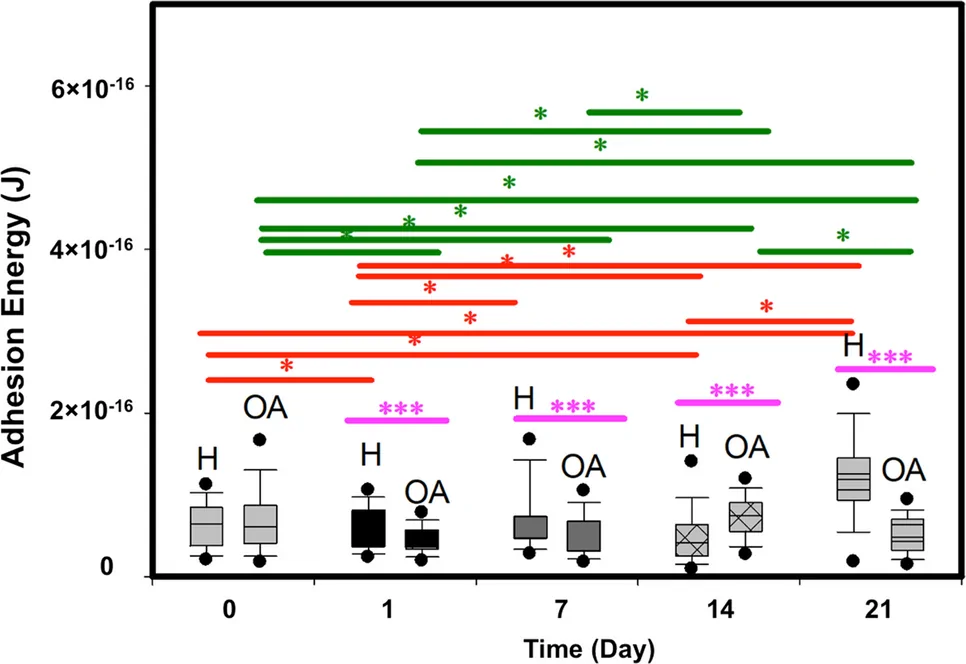
Adhesion energy levels of healthy (*H*) and osteoarthritis (*OA*) human chondrocytes over 21 d. Adhesion was measured at five time points (days 0, 1, 7, 14, and 21). Data are presented as box plots (median, interquartile range, and individual data points). The estimated marginal means ± standard error (SE) derived from the linear mixed model. This model accounts for the hierarchical nesting of technical replicates within individual donors (*N* = 3 per group). *One asterisk* indicates significance (*P* < 0.05), *three asterisks* significance (*P* < 0.001).

To investigate how adhesion strengths varied between healthy and OA donors, cell adhesion energies were measured for the three donors representing each phenotype. This data reflected 81 cells for each donor type, with AFM mapping yielding between 1555 and 1712 data points for each box plot shown in Fig. 3.

When comparing healthy and OA phenotypes, differences were observed at days 0, 1, 7, 14, and 21. The ratio of mean adhesion energies for all cells of healthy donors compared to OA donors varied by up to 1.1-, 1.3-, 1.3-, 1.3-, and 2.3-fold on days 0, 1, 7, 14, and 21, respectively (Table 2). This suggests that the adhesion strength of cells from healthy donors consistently exceeded that of cells from OA donors.

**Table 2.** Mean adhesion energies (E; × 10^–17^ J) for healthy (*H*) and osteoarthritis (*OA*) donors

	Day 0 (10^–17^ J)	Day 1 (10^–17^ J)	Day 7 (10^–17^ J)	Day 14 (10^–17^ J)	Day 21 (10^–17^ J)
Healthy	6.3 ± 2.9	6.0 ± 2.9	7.0 ± 4.2	5.4 ± 5.6	12.2 ± 5.7
OA	7.0 ± 4.5	4.6 ± 1.8	5.1 ± 2.9	7.3 ± 2.8	5.1 ± 2.5

Values are reported as mean ± standard deviation

These variations likely reflect changes in surface molecules and ECM interactions during culture. Regardless of the cell type used to initiate AC-like tissue growth, cells undergo developmental stages including secretion of ECM rich in hyaluronic acid and COL, cell aggregation, proliferation, and secretion of adhesion molecules such as N-cadherins [20].

The higher adhesion observed in cells derived from healthy donors compared to those derived from OA donors can also be attributed to interactions between the cluster of differentiation 44 (CD44) and hyaluronic acid HA [21]. In contrast, OA cartilage-derived cells have been shown to exhibit reduced integrin-mediated cell-ECM interactions. β1 integrin signaling is often impaired in osteoarthritic cartilage, which may contribute to the reduced adhesion forces observed in these cells [21]. A separate study using AFM also investigated adhesion force in chondrocytes from healthy and OA donors. Their results demonstrated that OA-derived cells exhibited lower adhesion forces, whereas healthy donor-derived cells displayed higher adhesion forces. This was attributed to the collective behavior of surface proteins and their expression patterns in OA cells, which interact less strongly with AFM [22].

The expression of integrins in chondrocytes, including α1β1, αvβ3, α2β1, α10β1, αvβ5, α5β1, and α6β1, facilitates binding to COL II, vitronectin, fibronectin, and laminin in cartilage. Integrin β1, which is abundantly expressed in chondrocytes, helps anchor the contractile actin cytoskeleton to the cell membrane. In OA cartilage, however, integrin β1-mediated cell-ECM interactions are reduced, suggesting that cell–matrix signaling disorders during OA contribute to decreased adhesion force [23].

### Chondrogenic marker quantification

**Figure 4.**
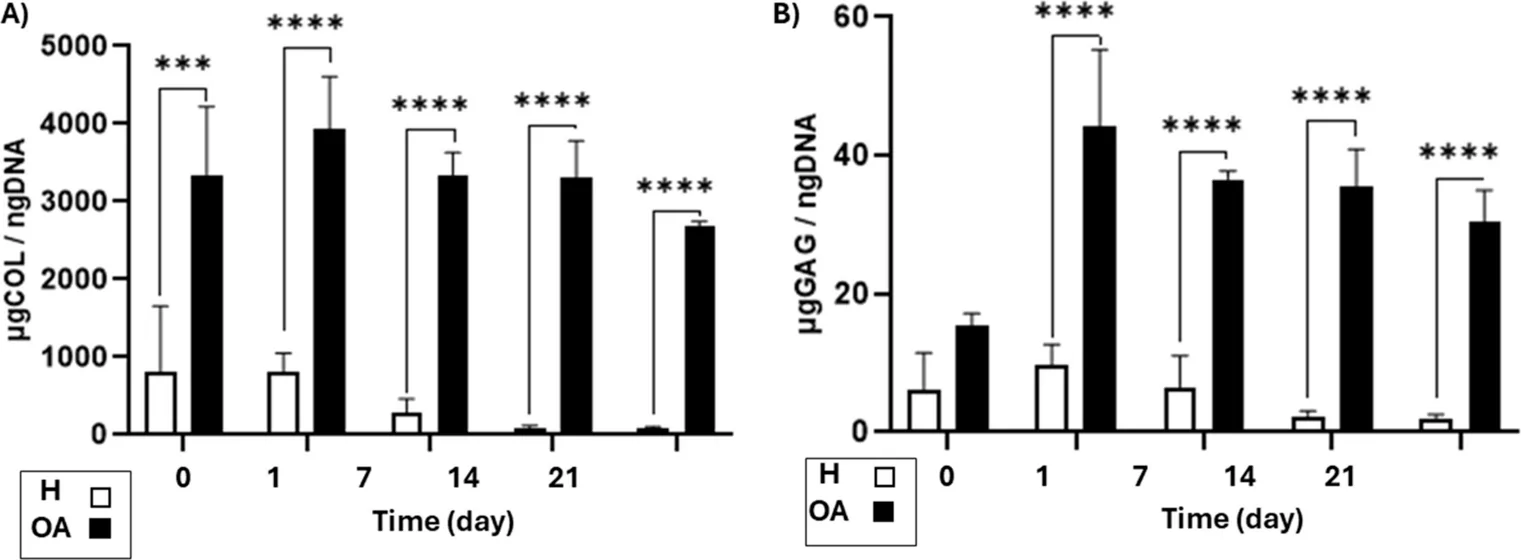
Biochemical characterization of the extracellular matrix (ECM). Quantified COL/DNA (*A*) and GAG/DNA (*B*) ratios for healthy (*H*) and osteoarthritis (*OA*) human chondrocytes at five time points (days 0, 1, 7, 14, and 21). Data are presented as estimated marginal means ± standard error (SE) derived from the linear mixed model (LMM), accounting for three independent donors (*N* = 3) and triplicate cultures per donor (*n* = 3). Donor identity was treated as a random effect to account for biological variability. *Three asterisks* indicate significance (*P *< 0.001), and *four asterisks *significance (*P* < 0.0001).

 COL produced by the chondrocytes isolated from healthy and OA donors are shown in Fig. 4*A* and Table 3 over a 21-d period while normalized by DNA. As illustrated, the OA-derived cells produced significantly more COL than the healthy cells at all culture time points (*P* < 0.001).

**Table 3.** Collagen (*COL*) and glycosaminoglycan (*GAGs*) concentrations normalized to DNA content (ng) for chondrocytes isolated from healthy (*H*) and osteoarthritis (*OA*) donors

	Day 0 (µg)	Day 1 (µg)	Day 7 (µg)	Day 14 (µg)	Day 21 (µg)
H-COL	816.0	807.0	289.0	89.0	83.0
OA-COL	3335.0	3926.0	3343.0	3318.0	2679.0
H-GAG	6.0	9.6	6.5	2.2	2.0
OA-GAG	15.0	44.0	36.5	35.0	30.5

Specifically, the ratios of COL secreted by OA cells relative to healthy cells were 4.1, 4.9, 11.6, 37.2, and 32.2 at days 0, 1, 7, 14, and 21, respectively (Table 3). Similarly, OA-derived chondrocytes showed significantly greater GAG production than healthy chondrocytes at all culture time points (*P* < 0.001).

These results suggest that OA-derived chondrocytes remain metabolically active and may increase ECM production as a compensatory response to cartilage destruction. COL is a primary protein in connective tissues, and its total amount and concentration in these tissues partially dictate their mechanical properties, including Young’s modulus of elasticity, tensile strength, and stiffness [24]. It should be noted that existing studies on COL types in OA are inconsistent; some report a decrease in COL II with an increase in other types, while others indicate an increase in COL II [25–27].

Chondrocytes derived from OA donors produce higher amounts of GAG than those from healthy donors. As shown in Fig. 4*B* and Table 3, OA-derived chondrocytes demonstrated significantly greater GAG production than healthy chondrocytes at all time points in culture (*P* < 0.001). Specifically, the ratios of total GAG in OA-derived cells compared to those isolated from healthy donors were 2.5, 4.6, 5.6, 15.9, and 15.3 at days 0, 1, 7, 14, and 21, respectively (Table 3). Similarly, increased GAG synthesis in OA-derived cells has been reported in several studies. This high production may represent a repair response to cartilage degradation aimed at maintaining ECM integrity [28, 29].

### Correlations between adhesion energy and ECM components

**Figure 5.**
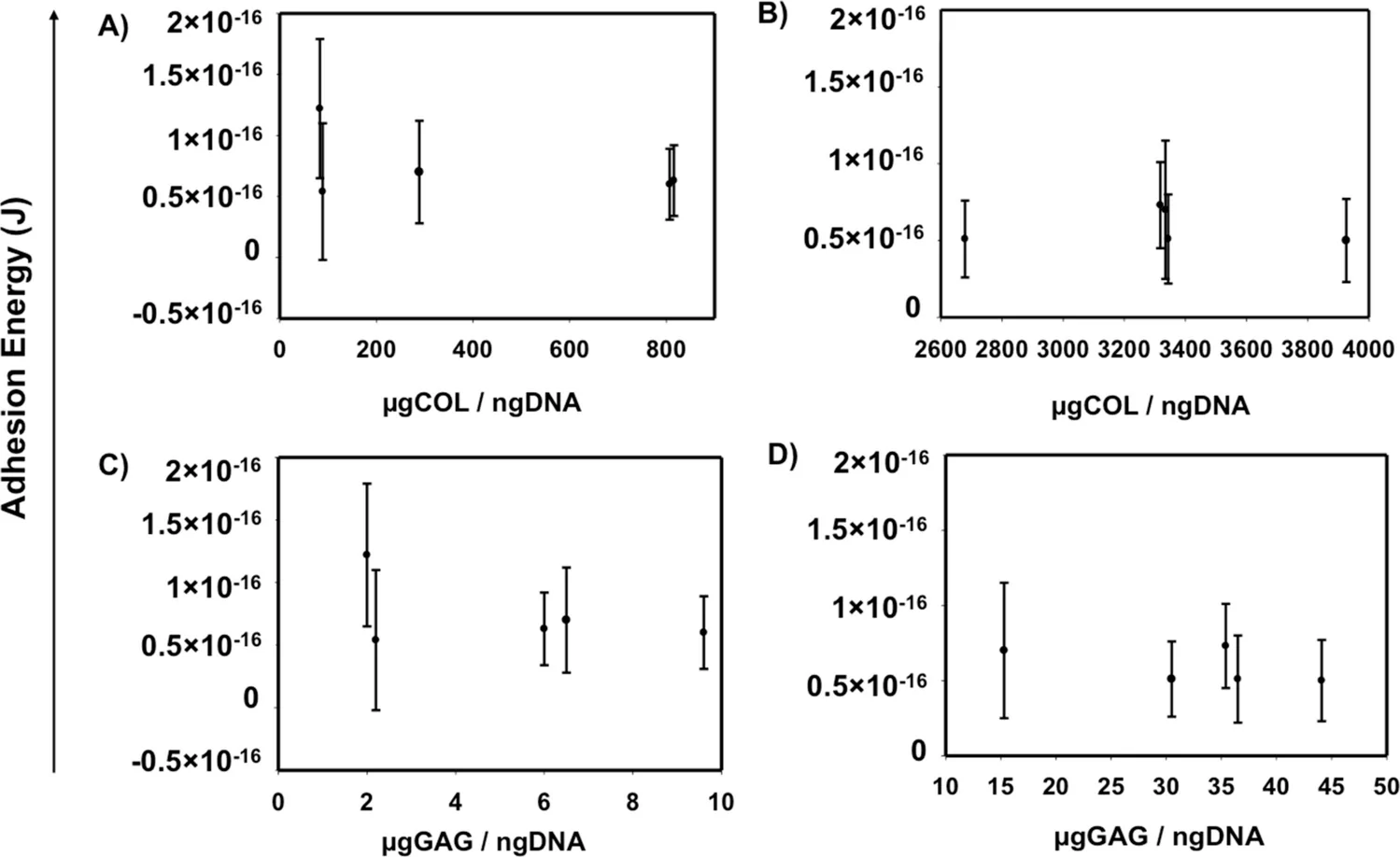
Correlations between adhesion energy and μgCOL/ngDNA for human chondrocytes isolated from (*A*) healthy (*H*) and (*B*) osteoarthritis (*OA*) donors and between adhesion energy and μgGAG/ngDNA in chondrocytes isolated from (*C*) healthy (*H*) and (*D*) osteoarthritis (*OA*) donors. *Error bars* represent the standard error of the mean. Triplicates were averaged and used for the corresponding donor.

The relationships between COL and GAG and adhesion energy between chondrocytes isolated from healthy and OA donors were explored. Our findings indicated that, irrespective of donor source, adhesion energy is independent of the COL content (Fig. 5*A* (*H*) and *B* (*OA*)). Figure 5*C* and *D* display the relationships between GAG and adhesion energy measured for chondrocytes isolated from healthy and OA donors across five different time points (day 0, day 1, day 7, day 14, and day 21), respectively. As evident from Fig. 5*C*, for chondrocytes isolated from healthy donors, no correlation was observed between adhesion energy and GAG. In contrast and shown in Fig. 5*D* for OA-derived chondrocytes, adhesion energy positively correlates with an increase in GAG content.

This positive correlation may be related to the role of GAG components such as chondroitin sulfate and hyaluronic acid in proteoglycans such as aggrecan, which influence the mechanical properties of cartilage and cell–matrix interactions. Chondrocytes interact with the ECM through receptors including integrins and CD44, which bind ECM molecules and transmit mechanical signals to the cytoskeleton. Changes in GAG composition or sulfation patterns can alter these interactions and affect adhesion energy [30, 31]. Such changes are often observed in OA, where ECM remodeling disrupts normal cell–matrix signaling [32].

## Limitations

While this study focused on male-derived cells to minimize hormonal and dimorphic changes, we acknowledge that sexual dimorphism plays a major role in OA. Future studies are needed to reveal whether the adhesion-energy correlations identified here are also reflected in the female population. In addition, the relatively small number of donors used in this study is a limitation. Although AFM provides many measurements per donor, a larger donor cohort would improve generalizability.

## Conclusions

Our results demonstrated that donor-to-donor variations in adhesion energies were significant. Specifically, chondrocytes isolated from healthy donors consistently exhibited higher adhesion energies compared to chondrocytes isolated from OA donors. Furthermore, the present study revealed that OA-derived chondrocytes produced significantly more COL and GAG per DNA than chondrocytes from healthy donors. Our findings also suggested that adhesion energy is largely independent of COL and GAG for chondrocytes from healthy donors. In contrast, adhesion energy was positively correlated with GAG in chondrocytes isolated from OA donors, but independent of the COL.

## Data Availability

Data availability is restricted due to ongoing research efforts and possible disclosures.
